# Influence of Breed on Tracheal Diameter and Endotracheal Tube Size Selection in Dogs: A Retrospective Analysis

**DOI:** 10.3390/ani16193089

**Published:** 2026-10-01

**Authors:** Jieun Kim, In Lee, Won-gyun Son, Inhyung Lee, Hyunseok Kim

**Affiliations:** 1 Department of Veterinary Clinical Sciences, College of Veterinary Medicine, Seoul National University, Seoul 08826, Republic of Korea; je@ian.kr (J.K.); carpeego@snu.ac.kr (W.-g.S.); inhyunglee@snu.ac.kr (I.L.); 2 Ian Animal Diagnostic Center, Seoul 06258, Republic of Korea; in@ian.kr; 3 Department of Veterinary Anesthesia and Pain Medicine, College of Veterinary Medicine, Konkuk University, Seoul 05029, Republic of Korea

**Keywords:** airway management, brachycephalic breed, body weight, computed tomography, intubation, South Korea, trachea

## Abstract

Selecting the correct endotracheal tube (ETT) size is critical for safe anesthesia in dogs. Current ETT size recommendations are largely based on body weight (BW), although some breeds, particularly brachycephalic dogs, may receive smaller ETTs than predicted from BW alone. This two-part retrospective study was performed to evaluate the effect of breed on ETT selection. In Part 1, computed tomography (CT)-measured tracheal diameters (TD) were compared among five small dog breeds weighing 4–5 kg and having a body condition score of 5/9. In Part 2, an expanded clinical cohort of 512 dogs from eight breeds was used to compare clinically selected ETT sizes with BW-based predictions. Statistical models assessed whether breed influenced clinically used ETT sizes after accounting for body mass using the cube root of BW. CT measurements revealed that TD differed significantly among breeds within a similar body-weight range. In the Part 2 clinical cohort, clinically selected ETT size was significantly associated with breed, the cube root of BW, and their interaction. Shih Tzus, French Bulldogs, Pekingese, and Boston Terriers had clinically selected ETT sizes smaller than BW-based predicted sizes, with a median discrepancy of approximately 0.5 mm in Shih Tzus and approximately 1.0 mm in French Bulldogs, Pekingese, and Boston Terriers. These findings suggest that the magnitude of discrepancy from BW-based predictions varies among brachycephalic breeds, indicating that breed may warrant consideration when estimating ETT size.

## Simple Summary

Selecting the correct endotracheal tube (ETT) size is critical for dogs and their safety. A tube that is too small may increase airflow resistance and cause gas leakage or inadequate airway protection, whereas a tube that is too large may complicate intubation and injure the airway. In clinical practice, tube size is often chosen based on the dog’s body weight (BW); however, dogs of similar weight may have different airway dimensions depending on breed. First, the authors used computed tomography scans to compare tracheal diameters among five small dog breeds with similar BW. Next, the authors reviewed records from 512 dogs across eight breeds to see what tube sizes were clinically used in practice compared with the sizes predicted from BW. Results from Part 1 showed that tracheal diameter varied among breeds even within a narrow BW range. In the separate Part 2 cohort, the relationship between body mass and clinically selected ETT size differed among breeds when body mass was expressed using the cube root of BW. In brachycephalic breeds, clinically selected ETT sizes were generally smaller than BW-based predicted sizes, and the magnitude of the discrepancy varied among breeds, suggesting that breed-related considerations may be useful when estimating candidate ETT sizes.

## 1. Introduction

Endotracheal intubation is a fundamental procedure in veterinary anesthesia, emergency medicine, and critical care [1]. Appropriate endotracheal tube (ETT) selection is essential for maintaining airway patency, providing effective ventilation, and minimizing complications associated with airway management. In general, the largest tube that can be inserted without resistance is recommended [2,3]. ETTs that are too small may increase anesthetic gas leakage and the risk of inadequate airway protection. According to Poiseuille’s law, airflow resistance increases as ETT internal diameter decreases; therefore, even small reductions in tube size may increase resistance to ventilation. ETTs that are too large may increase insertion difficulty and the risk of mucosal or pressure-related airway injury [1,2,3,4,5,6].

In small animal practice, ETT size is often estimated using body weight (BW), external anatomical landmarks, or operator experience [1,7,8]. Previous studies have also proposed BW-based methods for predicting appropriate ETT size [2,4,9]. Although BW-based methods provide a practical approach to ETT size estimation, adjustment may be needed in some breeds. In particular, previous studies have reported that brachycephalic dogs may require smaller ETTs than those predicted from BW alone [10]. In addition, several references recommend preparing ETTs in the expected size as well as one size smaller and larger in anticipation of mismatch during intubation [1,2,4,5,10]. However, repeated intubation attempts may increase laryngeal trauma, including iatrogenic laryngeal paralysis and tracheal mucosal injury with inflammation [11,12]. Therefore, considering breed in addition to BW may provide a more refined approach to ETT size selection before intubation.

To more comprehensively evaluate breed-related influences on airway size and clinical ETT selection, the present study was conducted in two parts. Part 1 was designed to determine whether CT-measured tracheal diameter differed among breeds even within a restricted body-weight range. Part 2 included dogs across a broader body-weight distribution to evaluate whether breed influenced clinically selected ETT size beyond the effect of body weight.

The authors hypothesized that tracheal size would differ among breeds, that the relationship between body mass and clinically selected ETT size would vary by breed, and that the magnitude of discrepancy between clinically selected and BW-based predicted ETT sizes would differ among breeds.

## 2. Materials and Methods

### 2.1. Study Design and Ethical Approval

This retrospective study comprised two independent analyses using separate cohorts with different inclusion criteria. The study protocol was reviewed and approved by the Institutional Animal Care and Use Committee of Seoul National University (IACUC No. SNU-250421-3).

### 2.2. Part 1: CT-Based Analysis of Breed-Related Differences in Tracheal Diameter

For Part 1, medical records of dogs that underwent CT examination at the Ian Animal Diagnostic Center between January 2020 and December 2024 were retrospectively reviewed. CT examinations were performed for clinical indications unrelated to primary respiratory disease, most commonly for tumor staging/metastatic evaluation or assessment of intra-abdominal disease, and were not performed specifically for this study. Dogs were included if they belonged to one of five small breeds commonly encountered in Korean clinical practice (Maltese, Poodle, Pomeranian, Shih Tzu, and Bichon Frise), weighed between 4 and 5 kg, and had a body condition score (BCS) of 5/9, as recorded in the medical record using the 9-point scoring system. Medical records were reviewed for a history or clinical evidence of respiratory disease, and CT images were reviewed for structural airway abnormalities and respiratory pathology. Dogs with incomplete records or any evidence of respiratory disease or structural airway abnormality were excluded.

Anesthetic management was performed according to routine clinical practice, and relevant data were obtained from the medical records. Dogs were typically fasted for at least 6 h before anesthesia, with free access to water until approximately 2 h before induction, according to institutional protocols. A 24-gauge intravenous catheter (0.7 × 19 mm; BD Angiocath Plus^TM^, Becton Dickinson Medical, Singapore, Singapore) was generally placed in the cephalic vein. Premedication most commonly consisted of butorphanol (0.2–0.3 mg kg^−1^; Butophan Inj., Myung Moon, Seoul, Republic of Korea) and midazolam (0.2–0.3 mg kg^−1^; Midacum Inj., Myung Moon, Seoul, Republic of Korea) administered intravenously. Before intubation, the intended ETT insertion depth was estimated by externally measuring the distance from the incisors to the point of the shoulder, with the aim of positioning the ETT tip near the thoracic inlet. Anesthesia was then induced with propofol (4–5 mg kg^−1^; Anepol Inj., Hana Pharm, Seoul, Republic of Korea). Dogs were intubated in sternal recumbency using cuffed endotracheal tubes (Rüschelit super safety clear, Teleflex Medical, Morrisville, NC, USA). Cuff inflation was performed using the minimum occlusive volume technique, with confirmation of an adequate seal at approximately 2 kPa (approximately 20 cmH_2_O). Anesthesia was maintained with sevoflurane (Sojourn Solution; Kyongbo Pharm, Asan, Republic of Korea) delivered in oxygen using a circle rebreathing system, with an oxygen flow rate of approximately 1 L/min. Standard intraoperative monitoring included heart rate, oxygen saturation, end-tidal carbon dioxide, and oscillometric arterial pressure.

CT imaging was performed as part of routine clinical practice, and acquisition parameters were retrieved from imaging records. Images were obtained from the tip of the nose to the diaphragm using a CT scanner (Somatom go.now; Siemens AG, Munich, Germany). The CT scanner underwent routine system calibration once daily at scanner startup before clinical imaging, in accordance with the manufacturer’s recommended procedure. CT dose index measurements were performed at least once every 6 months. During CT image acquisition, manual ventilation was applied, and short breath-hold periods were performed with airway pressure maintained at 2 kPa (approximately 20 cmH_2_O). Scan parameters showed minor variation among cases but were largely consistent, typically including 130 kVp, 90 mAs, a rotation time of 0.8 s, 1 mm slice thickness, and a helical pitch of 1.

**Figure 1:**
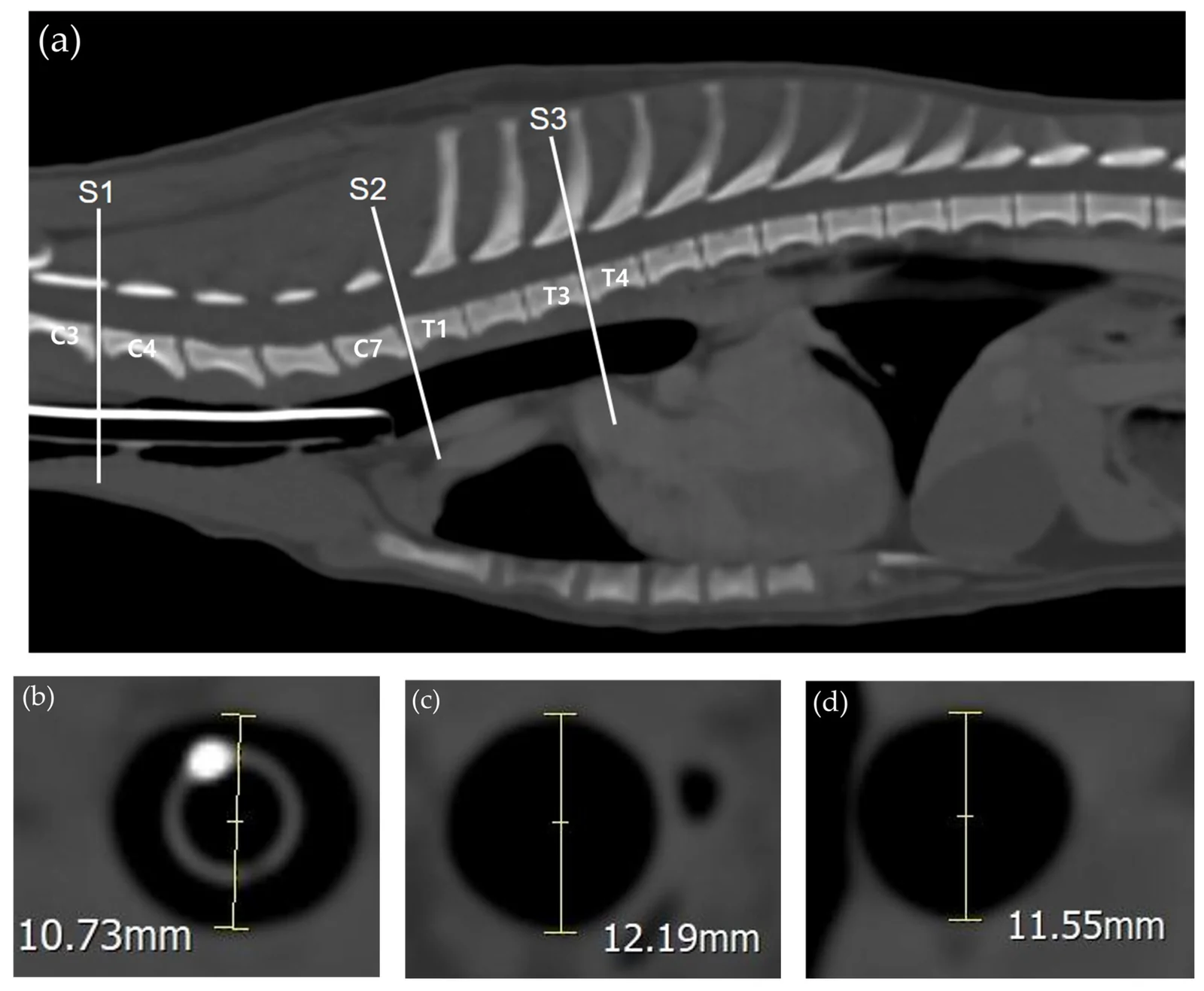
Computed tomography (CT) images showing the three tracheal measurement sites. The sites were identified on a sagittal CT image (**a**), and vertical luminal diameters were measured on transverse lung-window images at the cervical S1 (C3–C4; panel (**b**)), thoracic inlet S2 (C7–T1; panel (**c**)), and intrathoracic S3 (T3–T4; panel (**d**)) segments. C, Cervical vertebra; T, Thoracic vertebra.

The CT images were reviewed to confirm that the ETT tip was positioned near the thoracic inlet in all included dogs. Using clinical imaging data, TD was measured on transverse CT images at three anatomical levels: the cervical trachea (C3–4), thoracic inlet (C7–T1), and intrathoracic trachea (T3–4), as shown in Figure 1. The cervical measurement was obtained at the transverse plane through the midpoint of the C3–C4 intervertebral disc. The thoracic inlet measurement was obtained at the midpoint of the C7–T1 intervertebral disc, and the intrathoracic tracheal measurement was obtained at the midpoint of the T3–T4 intervertebral disc. At each level, the internal vertical luminal diameter was measured perpendicular to the long axis of the tracheal lumen.

### 2.3. Part 2: Clinical Analysis of Clinically Selected and BW-Based Predicted ETT Size

For Part 2, anesthetic records of dogs managed at the Ian Animal Diagnostic Center between January 2023 and December 2025 were retrospectively reviewed. The expanded clinical cohort comprised eight breeds: Maltese, Poodle, Pomeranian, Shih Tzu, Bichon Frise, French Bulldog, Pekingese, and Boston Terrier. To minimize the confounding effects of body condition, only dogs with a recorded BCS of 5/9 on the 9-point scoring system were included. Dogs were eligible if BW, BCS, and the ETT size used during clinical anesthesia were available in the anesthetic records. A review of the medical records confirmed that the patients in Part 2 underwent anesthesia using the same protocol as those in Part 1. Only one anesthetic record per dog was included in the analysis. ETT selection was performed by three clinicians using the same general clinical approach. The initial ETT size was estimated based on BW and routine clinical assessment. In brachycephalic dogs, a smaller initial candidate ETT was typically selected than would be expected from BW alone, consistent with previous evidence that BW-based estimates tend to overestimate clinically used ETT size in brachycephalic dogs [10]. Tube fit was subsequently evaluated according to ease of passage and adequacy of airway sealing. The leak test was performed using the same clinical criterion across clinicians, and an adequate airway seal at an airway pressure of approximately 20 cmH_2_O was considered acceptable. If marked resistance was encountered during insertion, a smaller ETT was selected, whereas if the leak test indicated an inadequate airway seal, the ETT size was adjusted upward as clinically appropriate.

The same ETT product as that used in Part 1 was used throughout Part 2 cohort. Clinically selected tube size was obtained from the anesthetic records. For each dog, the BW-based predicted ETT internal diameter was calculated using the equation reported by Haider et al. [10]: ETT size (mm) = 2.407 × ln[BW (kg)] + 1.820, where BW is body weight in kilograms. The unrounded predicted value was used for the primary statistical comparison with clinically selected ETT size. For descriptive clinical interpretation, the predicted value was additionally rounded to the nearest 0.5 mm internal-diameter increment, corresponding to the available size increments of the ETT product used in this study. To evaluate breed-related differences in the discrepancy between clinically selected and BW-based predicted ETT sizes, the Haider equation was applied to all breeds, including brachycephalic breeds, as a common BW-based reference rather than as a validated clinical recommendation. The discrepancy was calculated as follows:

*Discrepancy* = *clinically selected ETT size* − *unrounded BW-based predicted ETT size*.

### 2.4. Statistical Analyses

All statistical analyses were performed using SPSS version 29.0 (IBM Corp., Armonk, NY, USA). Data distribution was assessed using the Shapiro–Wilk test, and statistical methods were selected according to distributional characteristics of the data. Continuous variables including age, body weight, and TD are presented as mean ± standard deviation (SD). ETT size is presented as median (interquartile range). In Part 1, CT-measured TDs at the cervical, thoracic inlet, and intrathoracic levels were measured independently by two observers. Interobserver agreement was assessed using the intraclass correlation coefficient (ICC) based on a two-way mixed-effects model for absolute agreement. ICC values were reported with 95% confidence intervals (CIs). The mean value of the two observers’ measurements was used for subsequent statistical analyses. For the analysis of CT-measured TD, a linear mixed-effects model was used to account for repeated measurements obtained from the three anatomical sites within each dog. Dog was included as a random intercept, while breed, anatomical site, sex, age, and BW were included as fixed effects. A breed-by-site interaction term was included to evaluate whether breed-related differences in TD varied according to anatomical location. When a significant breed-by-site interaction was identified, estimated marginal means were calculated and pairwise breed comparisons were performed separately at each anatomical site with adjustment for multiple comparisons. Model results are reported as estimated marginal means with 95% confidence intervals, and a *p*-value < 0.05 was considered statistically significant.

In Part 2, a general linear model was constructed with clinically selected ETT size as the dependent variable, breed as a fixed factor, and the cube root of BW as a continuous covariate. To evaluate whether the relationship between the cube root of BW and clinically selected ETT size differed among breeds, a breed-by-cube root of BW interaction term was included in the model. Because the interaction was significant, breed-specific relationships between the cube root of BW and clinically selected ETT size were subsequently examined rather than assuming a common slope across breeds. Breed-specific BW distributions and ranges were also examined to assess the degree of overlap among breeds. Model assumptions were evaluated by visual inspection of standardized residuals plotted against predicted values for linearity and homoscedasticity, inspection of residual histograms and normal Q–Q plots for residual distribution, and examination of influential observations using Cook’s distance.

For each breed, clinically selected ETT size was compared with the unrounded BW-based predicted ETT size using the Wilcoxon signed-rank test. The raw *p*-values are reported. To account for the eight breed-specific comparisons, a Bonferroni-adjusted significance threshold of α = 0.00625 (0.05/8) was applied. For each breed, the paired discrepancy was additionally summarized using the Hodges–Lehmann estimate of location shift, with 95% confidence intervals obtained from 20,000 bootstrap resamples.

## 3. Results

### 3.1. Study Population

#### 3.1.1. Part 1 Cohort

A total of 1498 medical records were screened for Part 1. Of these, 1162 dogs were excluded because they did not meet the BW criterion, 256 because they did not meet the BCS criterion, 29 because of respiratory disease or structural airway abnormalities, and 8 because of incomplete medical records or CT images. Consequently, 43 dogs were included in the final analysis. All dogs weighed between 4 and 5 kg, allowing breed-specific comparison of CT-measured tracheal diameters within a relatively narrow BW range. Baseline characteristics are summarized in Table 1.

#### 3.1.2. Part 2 Cohort

A total of 2830 anesthetic records were screened for Part 2. Of these, 2168 records were excluded because they did not meet the BCS criterion, and 150 records were excluded because of incomplete anesthetic records. The final analysis therefore included 512 unique dogs. Unlike Part 1, Part 2 was not restricted to a narrow BW range, because the clinical analysis was intended to evaluate ETT size selection across the broader BW distribution encountered in routine practice. All dogs had a BCS of 5/9. Baseline characteristics are summarized in Table 2.

### 3.2. Part 1: CT-Based Analysis of Breed-Related Differences in TD

**Figure 2:**
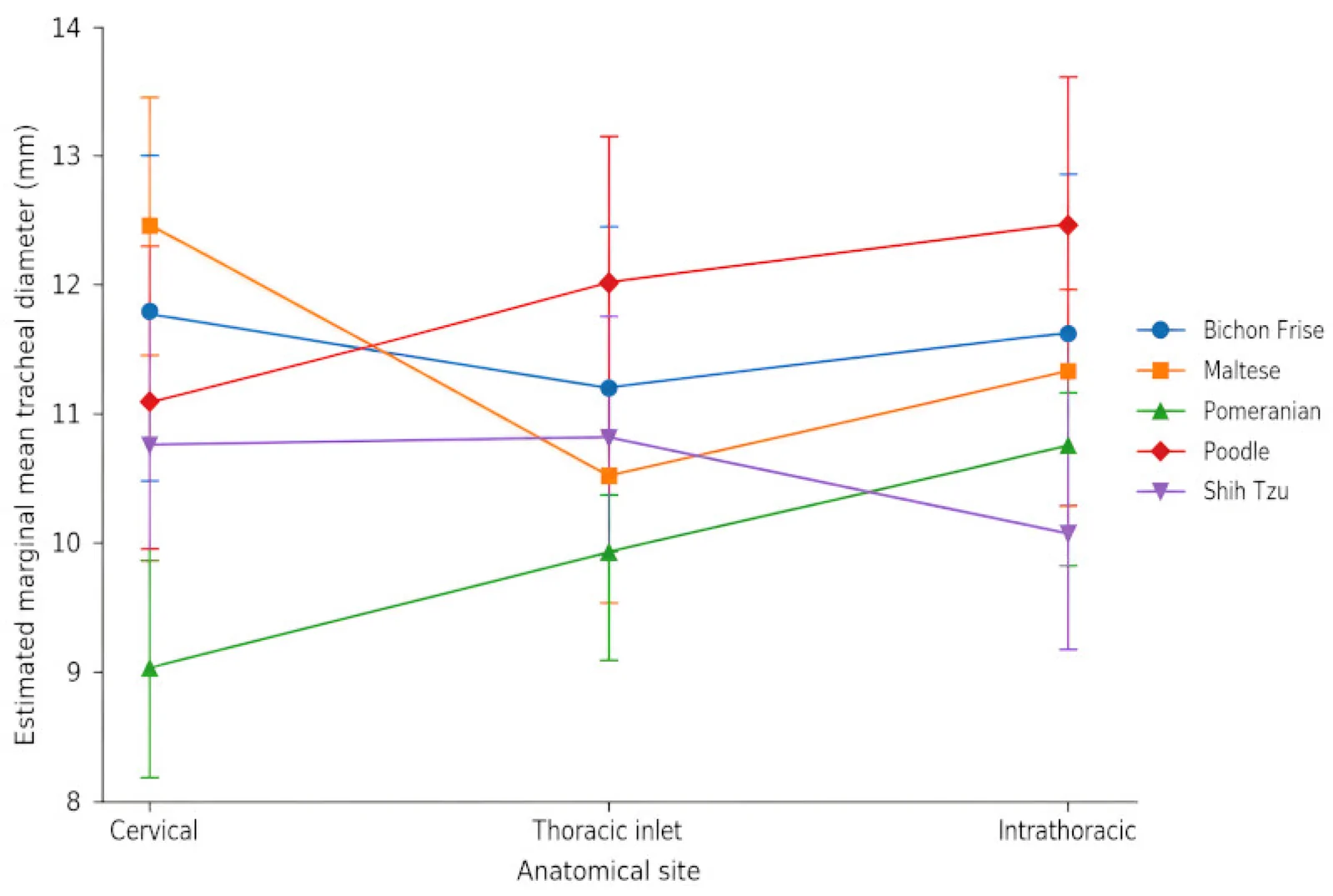
Breed- and site-specific differences in CT-measured tracheal diameter. Estimated marginal means adjusted for age, body weight, and sex are shown with 95% confidence intervals. A significant breed-by-site interaction was observed (*p* = 0.001).

In the linear mixed-effects model, breed was significantly associated with TD (F(4, 33) = 4.927, *p* = 0.003), whereas anatomical site alone was not significant (F(2, 76) = 1.007, *p* = 0.370). A significant breed-by-site interaction was identified (F(8, 76) = 3.705, *p* = 0.001), indicating that breed-related differences in TD varied according to anatomical location (Figure 2). Age (*p* = 0.105), BW (*p* = 0.764), and sex (*p* = 0.924) were not significantly associated with TD after adjustment in the model. Interobserver reliability was assessed using average-measures ICCs based on a two-way mixed-effects model with an absolute-agreement definition. The ICC values were 0.966 (95% CI, 0.314–0.991) for cervical TD, 0.831 (95% CI, −0.155–0.954) for thoracic inlet TD, and 0.752 (95% CI, −0.186–0.930) for intrathoracic TD. Site-specific pairwise comparisons following the significant breed-by-site interaction showed that, at the cervical level, TD was significantly greater in Maltese, Bichon Frise, and Poodle dogs than in Pomeranians (adjusted *p* < 0.001, *p* = 0.007, and *p* = 0.043, respectively). At the intrathoracic level, Poodles had a significantly greater TD than Shih Tzus (adjusted *p* = 0.031). No significant breed differences were identified at the thoracic inlet after adjustment for multiple comparisons.

### 3.3. Part 2: Effect of Breed on Clinically Selected ETT Size After Adjustment for BW

The general linear model including breed, the cube root of BW, and the breed-by-cube root of BW interaction was significant (F(15, 496) = 164.157, *p* < 0.001) and explained 83.2% of the variance in clinically selected ETT size (R^2^ = 0.832; adjusted R^2^ = 0.827). Breed (F(7, 496) = 5.503, *p* < 0.001), the cube root of BW (F(1, 496) = 251.549, *p* < 0.001), and the breed-by-cube root of BW interaction (F(7, 496) = 6.796, *p* < 0.001) were significant. The significant interaction indicated that the relationship between the cube root of BW and clinically selected ETT size differed among breeds. Breed-specific slopes relating the cube root of BW to clinically selected ETT size were 1.990 mm/kg1/3 (95% CI, 1.661–2.319) in Maltese, 3.049 mm/kg1/3 (95% CI, 2.713–3.385) in Poodles, 2.316 mm/kg1/3 (95% CI, 1.988–2.645) in Pomeranians, 4.216 mm/kg1/3 (95% CI, 3.415–5.016) in Shih Tzus, 3.253 mm/kg1/3 (95% CI, 2.948–3.557) in Bichon Frises, 2.643 mm/kg1/3 (95% CI, 1.620–3.665) in French Bulldogs, 2.257 mm/kg1/3 (95% CI, 0.301–4.212) in Pekingese, and 1.233 mm/kg1/3 (95% CI, −1.243 to 3.709) in Boston Terriers.Visual inspection of residual plots did not indicate substantial violations of linearity or homoscedasticity. The residual distribution was approximately normal, with mild deviations at the tails of the Q–Q plot, and no highly influential observations were identified (maximum Cook’s distance = 0.39).

### 3.4. Part 2: Comparison and Discrepancy Between Clinically Selected and BW-Based Predicted ETT Size

The magnitude of discrepancy varied substantially among breeds. The clinically selected ETT size matched the nearest commercially available 0.5 mm ETT size to the BW-based predicted value in 41.0–49.0% of Maltese, Poodle, Pomeranian, and Bichon Frise dogs. In Shih Tzus, exact match was lower at 25.5%, with a median discrepancy of −0.40 mm. Exact match was observed in only 2.0% of French Bulldogs, 0% of Pekingese, and 12.5% of Boston Terriers, in which the median discrepancies were approximately −1.0 mm. Differences of ≥1.0 mm occurred in 8.2–14.3% of Maltese, Poodle, Pomeranian, and Bichon Frise dogs and in 17.0% of Shih Tzus, compared with 85.7% of French Bulldogs and 75.0% of both Pekingese and Boston Terriers. Effect sizes were small in Maltese, Poodle, Pomeranian, and Bichon Frise dogs, but large in Shih Tzus, French Bulldogs, Pekingese, and Boston Terriers (Table 3). These findings indicate marked breed-related variation in the discrepancy, with Shih Tzus showing a less pronounced negative discrepancy than French Bulldogs, Pekingese, and Boston Terriers.

**Figure 3:**
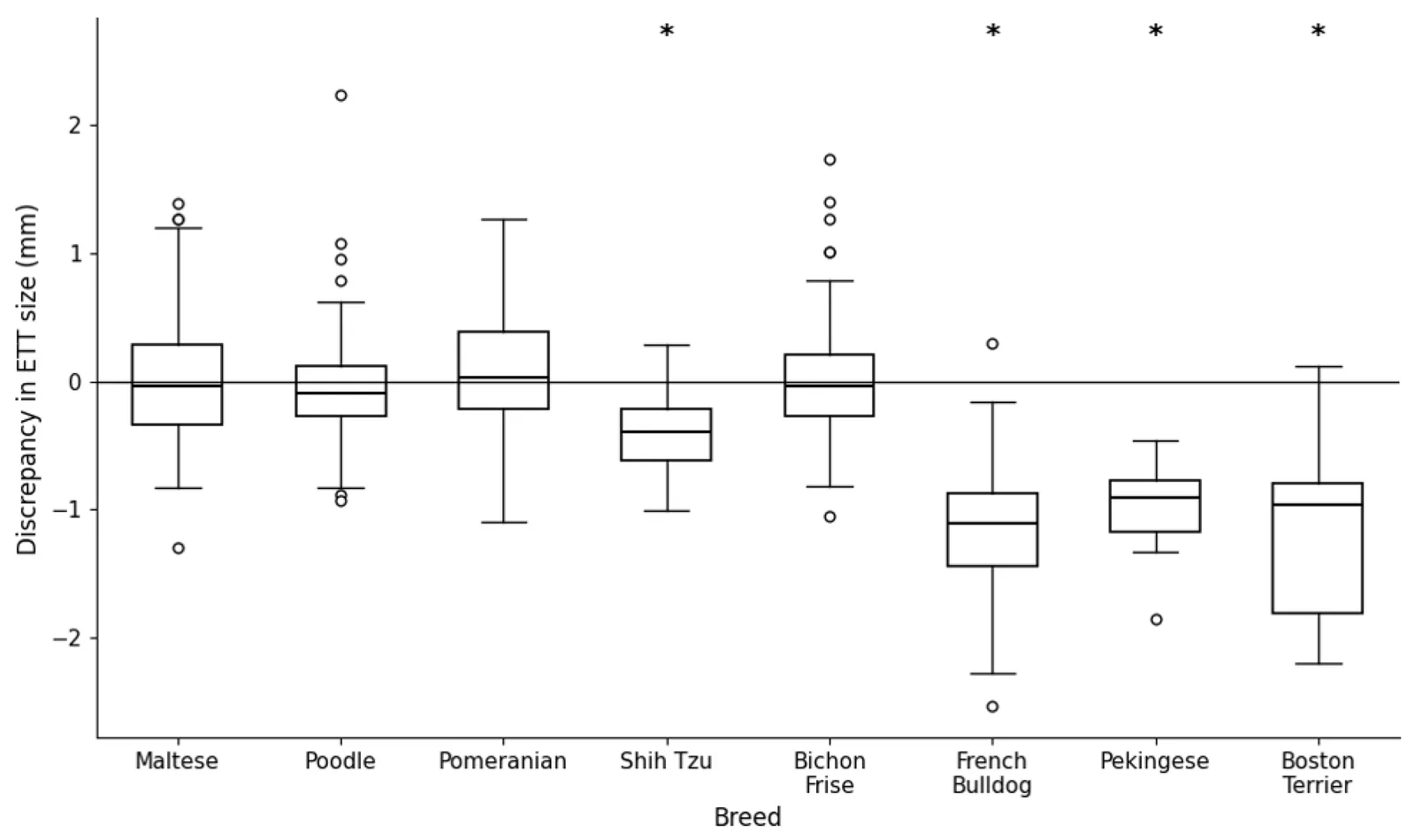
Box plots showing the discrepancy between clinically selected endotracheal tube (ETT) size and the unrounded body weight-based predicted ETT size by breed. Discrepancy was calculated as clinically selected ETT size minus the unrounded BW-based predicted ETT size. Values below zero indicate clinically selected ETT sizes smaller than the predicted value, whereas values above zero indicate larger clinically selected ETT sizes. Open circles indicate outliers. Asterisks indicate significant within-breed differences between clinically selected and BW-based predicted ETT sizes after Bonferroni correction using the Wilcoxon signed-rank test.

For statistical analyses, discrepancy was calculated as clinically selected ETT size minus the unrounded BW-based predicted ETT size. Negative values indicate that the clinically selected ETT size was smaller than the predicted value, whereas positive values indicate that it was larger. The distribution of these discrepancy values according to breed is shown in Figure 3.

## 4. Discussion

The present study comprised two related analyses designed to evaluate breed-related influences on ETT size selection in dogs. In Part 1, CT-based tracheal measurements demonstrated that TD differed significantly among breeds even within a similar BW range. This finding suggests that airway size may not be fully explained by BW alone and that breed-related anatomical variation may contribute to differences in tracheal dimensions. Differences observed at the cervical, thoracic inlet, and intrathoracic levels further suggest that breed-related airway variation extends along the tracheal course rather than being confined to a single segment. CT-based assessment was particularly useful because it allowed TD to be measured at anatomically defined levels without superimposition [13,14], thereby providing objective anatomical information for comparison across breeds.

In Part 2, breed, the cube root of BW, and the breed-by-cube root of BW interaction were significantly associated with clinically selected ETT size, indicating that the relationship between body mass and clinically selected ETT size varied among breeds. Because linear airway dimensions have previously been reported to scale with the cube root of BW [15,16], and the cube root of BW has also been evaluated as a predictor of ETT size in dogs [17], the cube root of BW was used to represent body-size scaling in the present study. The persistence of a significant breed-by-cube root of BW interaction suggests that breed-related differences in clinically selected ETT size were not explained solely by body-size scaling. In particular, significant discrepancies from BW-based predictions were observed in brachycephalic breeds, consistent with previous reports [10,18,19]. However, previous studies did not determine whether the magnitude of this discrepancy differed among individual brachycephalic breeds. In the present study, Shih Tzus showed a relatively small negative discrepancy, whereas French Bulldogs, Pekingese, and Boston Terriers showed substantially larger negative discrepancies. These findings suggest that brachycephalic breeds may not represent a homogeneous group with respect to the relationship between body weight and clinically selected ETT size. This distinction may be clinically relevant because brachycephalic dogs are particularly susceptible to difficult tracheal intubation owing to their characteristic airway anatomy. Previous reports have described greater resistance during tracheal intubation, as well as increased numbers of intubation attempts and longer intubation times, in brachycephalic dogs [20,21]. Greater resistance and repeated airway manipulation during intubation may increase the risk of airway injury, whereas delayed airway establishment may contribute to the development or worsening of hypoxemia. Therefore, selecting an appropriately sized ETT on the first intubation attempt may be particularly important in brachycephalic dogs to minimize repeated airway manipulation and facilitate timely airway establishment. The present findings indicate that the discrepancy between clinically selected and BW-based predicted ETT sizes may differ among brachycephalic breeds. Although further studies evaluating clinical outcomes are needed, these findings may support further consideration of breed-specific factors when refining ETT size-selection criteria for brachycephalic dogs.

This study has several limitations. First, the retrospective design may have introduced variability related to clinician judgment, prior experience, breed-related expectations, and ease of insertion. Although ETT selection was performed by three clinicians using the same general clinical approach and tube fit was assessed using a leak test, clinicians typically considered a smaller initial candidate ETT in brachycephalic dogs. Therefore, pre-existing breed-informed clinical selection practices may have contributed to the observed breed-related differences in Part 2. In addition, objective measures of anatomical fit, insertion resistance, post-intubation airway injury, and other clinical outcomes were not systematically available. Therefore, clinically selected ETT sizes may not necessarily represent anatomically optimal tube sizes. Second, Parts 1 and 2 were based on independent cohorts with different inclusion criteria. Therefore, CT-based tracheal measurements obtained in Part 1 cannot be directly matched to, or interpreted as a direct anatomical explanation for, the clinically selected ETT sizes observed in Part 2. Third, because CT measurements were obtained after endotracheal intubation, the ETT shaft and inflated cuff could have influenced tracheal geometry, particularly at the cervical and thoracic inlet levels. In addition, tracheal diameter alone does not determine the maximum ETT size that can be inserted clinically, because passage through the larynx may represent an additional anatomical limitation. Furthermore, the CT images used in this study were acquired at a 1 mm slice thickness with an in-plane pixel size of approximately 0.5–0.7 mm, and the geometric accuracy tolerance of the scanner was approximately ±0.5 to ±1.0 mm. Hence, the CT-derived TD values used for statistical analysis should not be interpreted as representing the absolute anatomical tracheal diameter with a level of precision corresponding to the reported decimal places. Fourth, interobserver reliability was generally high based on the ICC point estimates, although the wide confidence intervals indicate that these estimates should be interpreted cautiously. Finally, although breed-related differences in the discrepancy between clinically selected and BW-based predicted ETT size were statistically evaluated, these findings should be confirmed in prospective studies with larger and more balanced breed groups.

Despite these limitations, the present study provides complementary anatomical and clinical evidence that breed-related variation may be relevant to ETT size estimation beyond body weight alone. Breed-related effects appeared to be more evident among brachycephalic dogs. However, the heterogeneity observed among these breeds suggests that they may not constitute a homogeneous group for ETT size estimation. Further studies are needed to clarify the extent to which breed-related differences in clinically selected ETT size among brachycephalic dogs are associated with brachycephalic conformation and to identify objective measures that may help characterize these differences. Nevertheless, the present findings suggest that breed-related factors, particularly those associated with brachycephalic breeds, may warrant consideration in addition to BW when refining ETT size-selection approaches.

## 5. Conclusions

In the present study, although Parts 1 and 2 were based on separate cohorts, breed-related effects were identified in both airway dimensions and clinically selected ETT size. Consistent with previous reports, these effects were more evident in brachycephalic dogs, with the present study further demonstrating that the magnitude of breed-related differences varied among individual brachycephalic breeds. Because selecting an appropriate ETT on the first intubation attempt may be particularly important in brachycephalic dogs, these findings suggest that breed-related factors may warrant consideration when refining ETT size-selection criteria.

## Figures and Tables

**Table 1:** Baseline characteristics of dogs included in Part 1.

Breed	N	Age (Years)	Body Weight (kg)	Sex (MC/M/FS/F)
Maltese	9	11.4 ± 2.3	4.37 ± 0.32 (4.0–4.9)	6/1/2/0
Poodle	6	9.2 ± 2.9	4.52 ± 0.34 (4.0–5.0)	4/1/1/0
Pomeranian	10	9.7 ± 3.4	4.51 ± 0.31 (4.0–5.0)	4/1/4/1
Shih Tzu	11	11.0 ± 3.5	4.43 ± 0.34 (4.0–5.0)	1/0/7/3
Bichon Frise	7	3.3 ± 2.4	4.56 ± 0.33 (4.2–5.0)	1/0/4/2

All dogs had a body condition score of 5/9. Data of age presented as mean ± standard deviation, body weight presented as mean ± standard deviation (range), and sex distribution presented as counts. MC, castrated male; M, intact male; FS, spayed female; F, intact female.

**Table 2:** Baseline characteristics of dogs included in Part 2.

Breed	N	Age (Years)	Body Weight (kg)	Sex (MC/M/FS/F)
Maltese	100	9.5 ± 3.5	3.49 ± 1.09 (1.5–7.9)	46/4/45/5
Poodle	98	8.3 ± 3.8	4.61 ± 1.52 (1.2–9.6)	43/4/47/4
Pomeranian	112	6.7 ± 3.5	3.41 ± 1.09 (1.7–8.3)	50/3/49/10
Shih Tzu	47	11.8 ± 2.8	5.43 ± 1.02 (3.3–8.8)	19/0/26/2
Bichon Frise	74	5.9 ± 3.4	5.00 ± 2.17 (1.2–12.2)	38/0/29/7
French Bulldog	49	6.5 ± 2.5	11.57 ± 2.25 (7.8–18.1)	27/5/15/2
Pekingese	16	7.4 ± 4.3	5.84 ± 0.86 (4.6–8.1)	13/0/3/0
Boston Terrier	16	9.4 ± 3.5	9.81 ± 2.01 (6.2–13.6)	5/0/11/0

All dogs had a body condition score of 5/9. Data of age presented as mean ± standard deviation, body weight presented as mean ± standard deviation (range), and sex distribution presented as counts. MC, castrated male; M, intact male; FS, spayed female; F, intact female.

**Table 3:** Comparison between clinically selected and body weight-based predicted endotracheal tube (ETT) size within each breed.

Breed	Median Discrepancy (mm), IQR †	Hodges–Lehmann Estimate (95% CI), mm §	Exact Match, n (%) ‡	≥0.5 mm Difference, n (%) ‡	≥1.0 mm Difference, n (%) ‡	*p*-Value	Effect Size (r)
Maltese	−0.04 (0.62)	0.004 (−0.090 to 0.117)	41 (41.0)	59 (59.0)	14 (14.0)	0.912	0.01
Poodle	−0.10 (0.40)	−0.076 (−0.145 to 0.001)	48 (49.0)	50 (51.0)	8 (8.2)	0.058	0.19
Pomeranian	0.03 (0.60)	0.056 (−0.033 to 0.152)	51 (45.5)	61 (54.5)	16 (14.3)	0.261	0.11
Shih Tzu	−0.40 (0.44)	−0.414 (−0.511 to −0.334)	12 (25.5)	35 (74.5)	8 (17.0)	<0.001 *	0.84
Bichon Frise	−0.04 (0.50)	−0.018 (−0.108 to 0.087)	33 (44.6)	41 (55.4)	9 (12.2)	0.744	0.04
French Bulldog	−1.11 (0.59)	−1.152 (−1.282 to −1.019)	1 (2.0)	48 (98.0)	42 (85.7)	<0.001 *	0.87
Pekingese	−0.91 (0.44)	−0.959 (−1.127 to −0.816)	0 (0.0)	16 (100)	12 (75.0)	<0.001 *	0.88
Boston Terrier	−0.96 (1.09)	−1.138 (−1.503 to −0.825)	2 (12.5)	14 (87.5)	12 (75.0)	0.001 *	0.85

† Discrepancy was calculated as clinically selected ETT size minus the ETT size predicted using the body weight-based equation reported by Haider et al. Negative values indicate that the clinically selected ETT was smaller than the predicted size. ‡ For clinical interpretation, predicted ETT sizes were rounded to the nearest available 0.5 mm internal-diameter increment and the absolute difference from the clinically selected ETT size was categorized as exact match, ≥0.5 mm, or ≥1.0 mm. § Hodges–Lehmann estimates represent the paired location shift between clinically selected and unrounded BW-based predicted ETT size; 95% CIs were obtained using 20,000 bootstrap resamples. Raw *p*-values were obtained using the Wilcoxon signed-rank test comparing clinically selected ETT size with the unrounded BW-based predicted value. A Bonferroni-adjusted significance threshold of *p* < 0.00625 (0.05/8) was applied for the eight breed-specific comparisons. * Statistically significant at the Bonferroni-adjusted threshold of *p* < 0.00625. CI, confidence interval; ETT, endotracheal tube; IQR, interquartile range.

## Data Availability

The dataset has been uploaded and can be accessed at the following link: https://drive.google.com/drive/folders/1_9THxZVDgvw5SACgC1TCQYVfX97gNf7o?usp=sharing, accessed on 28 July 2026.

## Abbreviations

The following abbreviations are used in this manuscript:

BCS	Body condition score
BW	Body weight
CI	Confidence interval
CT	Computed tomography
ETT	Endotracheal tube
IACUC	Institutional Animal Care and Use Committee
ICC	Intraclass correlation coefficient
SD	Standard deviation
TD	Tracheal diameter
